# Supplementary material for: Are carbon emissions trading and green financial instruments synergistic? -Comprehensive quantitative research based on content analysis
Source: PLoS One. 2024 Mar 7;19(3):e0298601. doi: 10.1371/journal.pone.0298601 (PMC10919601; doi:10.1371/journal.pone.0298601)
Supplement: S1 Data — (ZIP) [file pone.0298601.s001.zip › Data/地级市Z_F工作报告.zip!/║┌┴·╜¡/─╡╡ñ╜¡╩╨/└┤╫╘╣1⁄2╓┌║┼í╛┬φ┐╦╩2╛▌═°í┐_2018.docx]

各位代表：

　　现在，我代表市人民政府，向大会报告工作，请予审议，并请市政协委员提出意见。

　　一、2017年工作回顾

　　2017年，在省委省政府和市委正确领导下，我们认真学习、全面贯彻党的十九大精神和习近平总书记对我省重要讲话精神，扎实推进全面振兴发展，经济社会保持稳中有进、稳中向好发展态势。预计全市地区生产总值增长6.5%，固定资产投资增长7%，一般公共预算收入增长11%，规上工业增加值增长3.5%，社会消费品零售总额增长9%，进出口总额增长25%，城乡居民人均可支配收入均增长7%。绥芬河、海林、穆棱、宁安、东宁保持全省十强县，林口居第18位，均实现位次前移。

　　一年来，我们主要做了以下工作：

　　（一）致力壮大实体经济，努力提升产业项目层次和实效，全面振兴发展基础更加坚实。持续开展“四比”活动和产业项目督导，狠抓落地开工、达产达效、园区提升和招商融资，新开工大基医疗健康示范基地、霖润天然纤维素等亿元以上产业项目113个、比上年增加28个；列入省重点的53个产业项目投资完成率达到113.5%；全市产业投资增长10%、占固定资产投资比重达到63%。百威英博30万吨啤酒、汇能光电、穆棱新凌万锭亚麻纱等40个项目形成新产能。各县（市）区为市经开区引入项目7个，市经开区在建10亿元以上产业项目5个、新签约13个，固定资产投资增长40%；7个重点园区完成投资155亿元。组建21个专业招商局，实施“五路并进”精准招商，成功举办CCTV“国家品牌高峰论坛”等活动，新引进艾诺威生物医药、绿色食药产业园等亿元以上项目80个，引进域外资金936亿元、增长10%，向上争取政策性资金超过180亿元；组建市投资集团，采取PPP模式融资5.2亿元，北味菌业在新三板挂牌。

　　（二）深入实施工业立市战略，扎实做好“三篇大文章”，工业基本面企稳回升。综合施策稳增长、调结构、转动能，规上停产企业由112户减少到12户，工业增加值由负转正、逐月回升。调控服务力度加大。全面落实减负降费政策，推出实体经济降成本增效益26项政策措施，取消行政事业性收费30项、为企业减负3864万元，为226户企业缓解资金紧张，为38户企业争取到省大用户直供电政策，我市获批国家增量配电业务改革试点；实施培育规上企业专项行动，全市新增规上企业33户。产业结构不断优化。实施“老字号”技改项目42个、完成投资21.5亿元，恒丰纸业设备改造升级等5个亿元以上技改项目投产达效，富通空调新增压缩机产能40万台，中车铸业产值增长1.7倍，友搏药业、首控石油等8户企业纳税超亿元，成为我市税源重要支柱；加快推进牡丹江烟厂、中鼎牧业液态奶加工、康师傅矿泉水二期等“原字号”项目，镈浩高纯石墨生产线投产，全市食品产业产值增长10%、矿产资源精深加工率达到20%；集中力量推进具有较大增长潜力和发展空间的“新字号”，珍宝岛药业、太敬机器人等项目超额完成年度投资计划，生物医药、高端装备制造等产业产值增速高于全市1.4个百分点。创新驱动深入推进。与中科院长春分院签署产业技术创新育成中心共建协议，牡丹江科技创新服务中心加盟机构、专家分别达到450家和808人，拥有高新技术成果2000多项，创新服务体系渐趋完善，我市成为国家知识产权试点城市；创新驱动效应开始显现，全市新增销售收入500万元以上科技型企业121户、科技企业孵化器12家，高新技术创业服务中心被评为国家级科技企业孵化器，全市高新技术产业增加值增长10.4%、高于全省平均和全市GDP增速3.9个百分点。民营经济加快发展。深入落实省支持民营经济发展若干意见，加大鼓励、支持、引导力度，我市成为国家民营经济发展改革示范城市。

　　（三）扎实推进农业供给侧结构性改革，加快一二三产业融合发展，“三农”工作再上新台阶。全市农业总产值突破400亿元，农民人均可支配收入有望实现全省“十五连冠”。以市场为导向加快结构调整。调减玉米种植50万亩、增加经济作物30万亩，粮食总产56.1亿斤；食用菌规模稳定在43亿袋（块）、标准化棚室突破1万栋，蔬菜面积达到51.5万亩，新增沙棘、大榛子等林下经济面积11.3万亩，北药面积增长1.9倍，畜牧业产值增长5.1%，绿色有机认证面积增加10万亩、达到478万亩。以龙头品牌营销为抓手提升产业化水平。突出“粮头食尾、农头工尾”建设，凯飞食品等8个亿元以上项目竣工，农业产业化龙头企业达到215户，宁安兰岗现代农业产业园、西安区台湾农民创业园成为国家级，绿色有机认证、国家地理标志产品分别达到205个和19个，农产品加工转化率达到47.8%；运用新模式、新业态助推农产品由“种得好”向“卖得好”转变，建设“互联网+农业”高标准示范基地96个、33.8万亩，签订众筹、点对点营销、集团定制、私人定制销售协议52项、实现收入2亿元，全市农产品电商销售额达到25亿元、增长39%。积极稳妥推进农村改革、完善服务保障体系。完成无纠纷争议农村土地确权颁证，基本完成农村集体资源性资产清产核资；设立农牧产业基金，村镇银行建设全省领先，农业承保面积占耕地51.9%；田间作业综合机械化率达到95%；农民专业合作社、家庭农场分别达到2253个和1264个。拓宽农民增收渠道。全面落实强农惠农政策，推动农民多渠道、多元化增收，农业旅游收入增长1.2倍，农民工资性收入增长14%，人均可支配收入有望突破1.7万元。

　　（四）增强消费对经济发展的基础性作用，持续释放需求潜力，现代服务业加快发展。第三产业对GDP增长贡献率达到60%，现代服务业比重达到49%、提高2个百分点。“旅游+”融合发展步伐加快。聚焦“两座金山银山”，高标准编制全域旅游规划及冰雪旅游规划，突出“好一派北国风光”主题，强化系统化集成营销，推出森林养生、文化体育等九大主题旅游产品，长汀至雪乡、林口至莲花公路通车，穆棱花海等5个亿元以上项目竣工，镜泊小镇启动运营，全市接待游客、实现收入分别增长22.6%和26.1%，我市成为中国十佳冰雪旅游城市；镜泊湖5A级景区整改通过国家验收，成功举办全国青少年“未来之星”冬季阳光体育大会、镜泊湖冬捕节等10大活动，接待游客跨越徘徊多年的50万大关、达到80万人次，旅游收入增长38%，人气加快集聚。新商业模式加快发展。总投资80亿元的月星环球城正式签约，远东国际商旅文小镇、江达城市综合体等新型商贸项目开工；加快国家级电子商务示范基地建设，建成本土电商平台11个，中国土特产网、农品天下网2个自建平台实现本地纳税，全市网络交易额突破140亿元、居全省前列；完成牡丹江机场货运监管场所建设，我市成为全国农产品冷链流通标准化示范城市。文化产业呈现新亮点。在巩固壮大传统文化产业的同时，向泛娱乐文化产业率先发力，与上海阅文集团签署战略合作协议，当当原创网络文学全国总部落户我市，全省第一个网络文学创意发展中心——牡丹江古道传媒投入运营，成功举办中国网络文学创作（牡丹江）峰会，我市成为国家文化消费试点城市。金融和房地产业保持平稳。新增金融机构4家、总数达到128家，金融业税收增长50%以上；市区商品房存量面积下降21.2%。

　　（五）积极融入“中蒙俄经济走廊”建设，加快发展开放型经济，沿边开放桥头堡作用进一步增强。以俄韩为重点的对外开放全面推进，全市口岸货运量首次突破1200万吨、出入境人数突破140万人次，对外贸易扭转连续37个月负增长态势。口岸通道更加畅通。绥东机场开工建设，哈牡客专、哈牡既有线电气化改造高效推进，与俄方草签建设东宁界河桥协定文本，绥芬河公路口岸联检大楼投入使用、铁路口岸获批汽车整车进口口岸，华晟物流入选全国多式联运示范工程、货运量超过45万吨，“哈绥符釜”陆海联运大通道与俄远东“滨海一号”实现对接，牡丹江机场客运量超过78万人次、迁建工作争取到空军支持。跨境产业加快发展。新增注册资金千万元以上外贸企业21户，俄粮回运加工、水产品加工等一批跨境产业项目加快建设，侨兴现代农业加工等4个项目投产运营，地产品出口、进口资源落地加工比重达到18%和54%；境外农业合作面积达到520万亩、占全省60%，东宁成为国家级“农业对外开放合作试验区”。平台建设取得重大突破。绥东重点开发开放试验区获国家资金支持和17项省级审批权限，牡丹江保税物流中心（B型）建成并获准运营，华信中俄现代农业产业园区享受俄远东跨越式发展区政策；全市跨境包裹邮递量增长85.6%、跨境人民币结算量超过11亿元。对外交流不断拓展。推进文化体育、教育科技、医疗卫生等领域全方位交流合作，成功举办第七届亚欧地区国际标准舞大赛等活动，与俄罗斯普希金俄语学院、远东联邦大学建立高中赴俄留学通道和教育交流基地。与东莞市签订对口合作协议，“五个一”活动务实有效开展。

　　（六）统筹推进城乡和生态文明建设，大力改善城乡面貌，国际现代山水森林城市建设取得新进展。坚持以人为核心，提升功能和品位，城乡空间布局和人居环境明显改善。中心城区基础设施建设高效推进。重新修订的《城市总体规划（2006-2020年）》获国务院批准，江南新城城市设计等12个专项规划启动编制。顺应百姓需求，打通“断头路”、“卡脖路”，畅通城市“微循环”，老城区18项新建路桥工程通车、新建整修人行道70条，江南新城新建续建道路13条，西十一公铁立体交通工程、西环江路和牡丹江大桥两侧匝道竣工通车，全面形成跨江、跨铁、环江、环城的立体化路网体系，城市交通更加顺畅，百姓出行更加快捷；完成新火车站南站房立面装饰亮化和光华街部分建筑立面改造，再造中西合璧新地标；改造燃气管网49公里、新增天然气用户17万户，结束了市区管道煤气使用历史。江南新城承载功能显著增强。“六横七纵”交通路网基本实现区划内园区互联互通，4所新建学校主体封闭，中医院江南分院、体育健身走廊投入使用。全国文明城市创建取得阶段性成果。我市在123个地级以上提名城市中综合排名跃升15位、进入第一方阵。全面开展城市环境整治“四季行动”，绿色清雪获央视点赞，市区新增绿地30万平方米、免费公共停车泊位1785个，更换路牌1500座，我市成为国家“十三五”首批公交都市创建城市。生态文明建设取得新成效。深入开展“减霾卫蓝、治污净水、保护农田”专项行动，中央环保督察组督办的高污染企业关停和近百户企业清洁排放改造工程全部完成，建成区淘汰燃煤锅炉441台，取缔黄标车4426辆，超额完成省下达主要污染物减排任务，万元GDP综合能耗下降3.5%；建立市县乡村四级“河长”体系，完成市区河湖水系连通治理年度任务和生活饮用水水源地一级保护区隔离防护工程，林海水库获水利部行业批复；全年植树造林8万亩、治理水土流失36万亩，落实农业“三减”示范田60万亩。新型城镇化和美丽乡村建设扎实推进。八面通等4个村镇被评为全国文明村镇，阜宁镇、下城子镇获批全国特色小镇，温春镇成为省经济发达镇行政体制改革试点镇，勤劳村、中兴村入选“中国少数民族特色村寨”，建设美丽乡村示范村47个，全省美丽乡村现场会在我市召开。

　　（七）坚持人民至上发展理念，尽心竭力保障和改善民生，让改革发展成果更多惠及全市人民。30件利民实事完成年度任务。全市民生支出突破230亿元、占一般公共预算支出比重达到83.9%，是民生投入数额最大、比重最高、覆盖面最广的一年。扎实推进精准扶贫精准脱贫。解决了628户贫困户住房难题、283户贫困户饮水质量安全问题，308名贫困人口实现脱贫。以大众创业万众创新拓展就业空间。集成120条优惠政策持续推动科技人员、大学生、农民、城镇转移就业职工“四支队伍”创新创业，全市众创空间达到30家，新增小微企业等市场主体2.9万户、增幅高于全省7.4个百分点，新增城镇就业5.6万人，城镇登记失业率控制在3.5%以内。加强基本生活保障。将自由职业者纳入住房公积金服务范围，改造棚户区8465户、农村“四类”人员危房1213户；五项社保新增参保5.2万人，基本完成城乡居民医保整合，异地就医实现直接结算，市区最低工资月标准提高180元，城乡低保标准实现十一连增，企业退休人员基本养老金实现十三连增。统筹推进社会事业。8个县（市）区义务教育均衡发展通过国家验收，高考上线率居全省前列。实施“健康牡丹江”行动，全面启动市级公立医院改革，全部取消药品加成；新增养老机构9个、床位1102张，为14万名60周岁以上老年人免费体检；“中西名医引进计划”邀请564名专家开展诊疗7000余人次；全民健身活动广泛开展，成功举办首届“健康雪城”冬季运动会。加强文化惠民，全面启动公共文化数字服务平台建设，地面卫星数字电视开播。加强社会治理创新。推进社区管理服务综合信息平台建设，城乡社区达标率达到88%和44%；扎实推进民族团结进步示范市、质量强市示范市创建；启动公共安全视频监控联网“雪亮工程”，加快完善社会治安防控体系，刑事案件、治安案件同比下降15%和6%、城区有19天实现刑事案件零警情；持续加强安全生产监管，安全生产事故起数、死亡人数分别下降20%和18.8%；深入开展“矛盾大排查、积案大起底、领导大接访”专项行动，化解疑难信访积案101件，8万户房产办证难和6000户逾期未回迁问题得到有效解决，党的十九大等重要节点进京非正常访登记全省最少。

　　各位代表，今年我市遭遇了1991年以来最大洪水侵袭，在市委正确领导下，全市上下紧密配合、协同作战，及时转移人员14147人，无一人伤亡，确保了群众安全、民心安宁、社会安定，取得了抗洪救灾的全面胜利。实践证明，只要我们团结一心、众志成城，就没有战胜不了的困难。

　　（八）围绕转变职能、提升效能，加强政府自身建设，施政能力和服务水平得到提升。“放管服”改革深入推进。建立公共服务、中介服务清单和政务服务事项目录，全面实行负面清单，取消行政审批事项39项，清理规范中介服务161项，推行“多证合一”企业登记模式，企业商事登记审批时限由1小时减至30分钟，367个行政许可及确认事项实现网上办理，“一屏妥”和送照上门分别占新办理件数、新注册企业的8%和20%。管理体制机制持续理顺。绥芬河重新划归牡丹江管理；镜泊湖管理体制问题纳入省深改组研究推进；绥东国家重点开发开放试验区管理体制调整到位；在全省率先成立行政审批局、综合行政执法局和行政监督局，条块式管理开始向“一章审批、一队执法、独立监督”平台式综合管理转变；全面完成事业单位分类，撤并事业单位19个，完成承担行政职能事业单位改革省级试点年度任务。法治政府建设取得新进展。配合市人大完成我市首部地方性法规——牡丹江市住宅物业管理条例立法工作，“双随机一公开”政府监管模式实现全覆盖；完善市政府“工作规则”和“议事规则”，全面推进政务公开和电子政务建设，自觉接受人大政协监督，议案、建议和提案办复率达到100%。作风和环境建设不断加强。深入开展机关作风整顿、党员干部大走访等活动，推动“两学一做”学习教育常态化制度化；加强政府审计监督，强化行政监察，从严正风反腐、肃清流毒；将16家单位迁至江南集中办公，腾退面积5.4万平方米，增收节支近亿元；严格执行“宁静工程”和“门禁制度”，深入开展“治慵懒、治梗阻、治推托”专项行动，查处损害和破坏发展环境案件50件。

　　统计、老龄、气象、地震、档案、人防、边防、民兵预备役、妇女儿童、残疾人、红十字等工作都取得新成绩，驻牡中省直部门、驻牡部队为地方发展做出突出贡献。

　　各位代表，过去一年，是极不平凡、极具挑战的一年。我们坚决贯彻市委部署，紧紧依靠全市人民，在增速换档中稳中求进、在结构调整中扬长补短、在深化改革中努力攻坚、在利益调整中兼顾公平、在统筹发展中注重协调、在矛盾多发时全力维稳，努力推动牡丹江全面振兴发展行稳致远。

　　成绩来之不易，这是省委省政府正确领导、关怀支持的结果，是市委总揽全局、科学决策的结果，是市人大市政协有力监督、帮助支持的结果，是全市人民同舟共济、不懈拼搏的结果。我代表市人民政府，向全市人民，向人大代表、政协委员，向离退休老领导、老同志，向各民主党派、工商联、无党派人士和人民团体，向中省直部门、驻牡部队、武警官兵和公安干警，向所有关心支持牡丹江发展的各界人士和国际友人，表示衷心的感谢！

　　同时，必须清醒地看到，我们的工作还存在许多不足，发展还面临不少困难和问题：一是经济高质量发展基础不牢。农业有特色但规模不够，一二三产有融合但不够充分；工业有规模但竞争力弱；现代服务业有成长但比例不高，开放生态资源有优势但释放不足，推动经济发展质量变革、效率变革、动力变革任重道远。二是民生高质量保障能力不足。城市建设有欠账、公共服务有短板、民生事业有差距，生态环境改善任务十分艰巨，解决人民日益增长的美好生活需要和不平衡不充分的发展之间的矛盾任重道远。三是政府高质量服务水平不够。部分政府干部解放思想浮于表面，市场化思维欠缺，专业能力、落实能力、基层组织力和群众动员力欠缺，有些工作进展低于预期，解决上述问题既任重道远更时不我待。对此，我们要强化问题导向，加倍努力工作，在今后的发展中认真加以解决。

　　二、2018年重点工作任务

　　2018年是贯彻党的十九大精神开局之年，是改革开放40周年，是决胜全面建成小康社会、实施“十三五”规划承上启下的关键一年，我们与全国人民同步进入了中国特色社会主义新时代。我们一定要清醒认识新时代的历史方位，努力把握新时代的重大历史机遇，聚焦解决新时代的主要矛盾，聚力建设现代化牡丹江的宏伟目标，奋力开创全面振兴发展新局面。

　　政府工作的总体要求是：深入学习贯彻党的十九大精神及中央经济工作会议精神，以习近平新时代中国特色社会主义思想为指导，坚持稳中求进工作总基调，坚持新发展理念，统筹推进“五位一体”总体布局，协调推进“四个全面”战略布局，全面落实“七大战略”，按照省委建设“六个强省”和打好“六个攻坚战”总体部署，市委“五个必须始终坚持”、打好“三大战役”要求和实现“九个新提升”目标，统筹推进稳增长、促改革、调结构、惠民生、防风险各项工作，推动经济社会持续健康发展，决胜全面建成小康社会，加快建设富强民主文明和谐美丽的现代化牡丹江。

　　经济社会发展预期目标是：地区生产总值增长7%左右，固定资产投资增长7%左右，一般公共预算收入增长5%，规上工业增加值增长5%，社会消费品零售总额增长8%左右，外贸进出口总额增长10%左右，城乡居民人均可支配收入均增长7%左右，万元GDP综合能耗下降3.2%，主要经济指标增速保持全省前列。

　　着重抓好以下七个方面工作：

　　（一）深化供给侧结构性改革,大力发展实体经济。实体经济是经济发展的根基。我们要按照“投资有回报、产品有市场、企业有利润、员工有收入、政府有税收、环境有改善”的要求，推进产业项目建设、创新驱动和新增长领域培育紧密结合，坚决打好转方式调结构攻坚战，加快建设现代产业体系。一是强化产业项目建设。加大项目督导、领导包保力度，持续保持对产业项目建设的紧逼加压态势，新开工亿元以上产业项目100个、10亿元以上工业项目10个，产业投资增长10%以上，珍宝岛药业加工厂、红豆杉万锭亚麻纺织、海川电气成套设备制造等45个项目竣工。强化市经开区龙头带动作用，年内新开工亿元以上产业项目30个。坚持“五路并进”抓招商，全年引进亿元以上项目100个。二是加快推进工业结构调整。坚持二产抓提升。突出“烟酒药乳水”等税源型产业、“三医三生三智”等高端产业，以食品、医药为重点，推动产业集群发展，夯实工业立市根基。实施“老字号”提升专项行动，因企施策、精准帮扶，大力支持传统企业技术改造、嫁接重组、裂变聚变，加快推进牡丹江烟厂、富通空调新品等重点项目建设；突出头尾衔接，延长产业链、提升价值链，加快推进红星乳业产业园、奥丰石墨深加工等“原字号”项目建设；围绕培育壮大“大智移云”等战略性新兴产业，加快推进法国欧亚机器人智能焊接、佳美3D打印材料、大衍车联网等“新字号”项目建设；全年工业投资保持全省前列。深入开展规模以上工业企业培育专项行动，鼓励和推进“高专特新”中小企业发展，支持民营企业发展，推动工业提质增效。三是加快发展现代服务业。坚持三产抓拓展。突出镜泊湖和中国雪乡两大品牌、推动“旅游+”融合发展、强化系统化集成营销，坚决打好旅游升级战役。加快镜泊湖综合开发、健康旅居养老示范基地、保利泛娱乐文化小镇、北京华星辉煌体育场馆等“旅游+”融合项目建设，丰富镜泊胜景、渤海古国、林海雪原等特色资源文化内涵，抓好旅游产品开发和旅游市场综合整治，突出“好一派北国风光”主题，创新旅游营销模式，全市接待游客、实现收入均增长20%以上，“候鸟养老”突破20万人次，文化产业增加值增长10%。加快新型商贸、金融、房地产业发展，推进月星环球城、远东国际商旅文小镇等重点项目建设，打造“不夜街”等特色街区，扶持本地纳税电商平台发展，全市电子商务交易额增长15%以上；抓好金融强市工作，落实服务实体经济、防控金融风险、深化金融改革三项重点任务，特别要将防控和化解政府性债务风险作为重中之重，推动农信社改制商业银行，加快佰佳信、红星乳业等企业上市进程；促进房地产业平稳健康发展。四是深入推进创新驱动和创新创业。落实创新驱动发展战略，加快国家知识产权试点城市建设，实施“科技型企业提质工程”，落靠抓实孵化器和众创空间三年行动计划，创建国家级高新技术产业开发区，全市新增国家级高新技术企业5户、科技企业孵化器和众创空间各10家、高新技术产业增加值增速高于全省平均水平。实施“百千万培训计划”，重点推动“四支队伍”创新创业，培训企业家300人以上、培养高技能人才3000人以上，加快建设知识型、技能型、创新型劳动者大军。

　　（二）坚持农业农村优先发展，全力做好新时代“三农”工作。我们要深入落实乡村振兴战略，扎实推进农业供给侧结构性改革，坚持质量兴农、绿色兴农，农业从增产导向向提质导向、进而向增效导向转变。一是以加快发展特色高效精品农业为重点调优农业结构。以市场为导向调减玉米10万亩发展经济作物，新增以“一耳双菇”为重点的棚室菌5000万袋、以“两果一药”为重点的林下经济12万亩、以“南销外运”为重点的蔬菜基地2万亩，以“两黑两白”为重点的精特畜牧业比重达到10%以上，扩大国家级绿色食品原料基地规模，新增绿色有机认证面积3万亩、绿色有机认证产品20个。二是以促进融合发展为重点加快建设现代农业产业体系。围绕把食品和农副产品精深加工业做成第一支柱产业的目标，加快越邦食品、宁安关东大厨房等“粮头食尾、农头工尾”项目建设，全市新增农业产业化龙头企业10户，农产品加工转化率再提高2个百分点，畜牧业加工产值增长10%以上；加大农产品品牌整合力度，维护好品牌形象，进入省农产品质量安全追溯公共服务平台绿色有机食品企业达到50%以上；按照“少卖水稻、多卖大米”的思路，统筹用好农产品营销“16条”措施，拓展应用众筹订制等新模式，发挥“农民电商讲习所”作用，利用好现有电商平台，鼓励经营主体自建电商平台，全年线上农产品销售额增长20%以上；加快建设农林产品、北药集散市场。三是以提升综合生产能力为重点加快建设现代农业生产体系。持续推进高效节水灌溉、中小河流治理等农田水利设施建设，启动建设10个以上现代农业科技示范园，新增节水灌溉面积2.5万亩，培训农民20万人次以上。四是以培育壮大新型经营主体为重点加快建设现代农业经营体系。探索小农户融入现代农业发展的实现形式，组建改建一批以农民土地、资金、技术入股为主要形式的利益联结紧密型合作社，大力支持同类合作社强强联合、企业与合作社产业链组合，全市规范合作社达到260个。五是以激活市场、要素为目标进一步深化农村改革。落实国家农村土地“三权分置”意见，完成土地确权工作，积极推进土地适度规模经营；加快农村集体产权制度改革，力争两年内完成全部改革任务。

　　（三）深度融入“中蒙俄经济走廊”建设，加快构筑全面开放新格局。开放是我市机遇、优势和希望所在。我们要以对俄日韩为重点，打好开发开放战役，加快培育经济合作和竞争新优势，当好全省“打造一个窗口、建设四个区”的排头兵。一是加快重大平台建设。举全市之力支持绥东国家重点开发开放试验区建设，加快推进规划编制、设施建设、政策落实争取和招商引资工作，努力在经济管理、要素集聚、投资贸易、产能合作等方面形成可复制可推广经验；牡丹江保税物流中心（B型）正式运营，并在此基础上争建牡丹江综合保税区、跨境经济合作示范区，力争我市纳入黑龙江（中俄）自由贸易区；支持境外园区提挡升级。二是加快集疏运体系建设。突出绥芬河、辐射牡丹江、放眼黑龙江，高水平编制集疏运体系规划。辟建绥芬河集疏运口岸中心，推动“哈绥符釜”陆海联运规范运营，畅通集铁路、公路、航空于一体的国际经贸大通道；辟建牡丹江物流园区，统筹各种运输方式协调发展，推进华晟物流多式联运示范工程建设，提升内陆港保税及仓储功能，打造“中蒙俄经济走廊”重要国际交通节点和国际物流集散枢纽。辟建牡丹江集疏运枢纽平台，依托云计算、车联网、卫星遥感等现代技术，探索建立综合运行保障机制，推动公路口岸、铁海联运、临空港区、信息平台一体化发展，逐步实现各物流节点交通互联、信息共享、业务互通、设施共用。2018年，要加快跨境电子商务产业综合服务平台、牡丹江快递监管中心等平台和绥东机场、牡佳客专、牡丹江机场迁建、东宁界河桥等重大基础设施项目建设，确保哈牡客专、哈牡既有线电气化改造按期通车，力争牡沈客专（牡丹江至敦化段）项目早日纳入国家铁路建设施工计划。三是大力发展外向型产业。围绕发展壮大食品深加工、石化煤化、林木林纸、轻工建材等跨境产业集群，扩大木材及对俄合作项下的大宗农产品进口规模，加快引进一批综合实力强、带动作用大的外贸加工企业，不断提高跨境产业层次。四是扩大对外交流合作。深化与国际友城合作，推动师生学习交流等工作，开展中医药诊疗和健康旅游等服务，市场化、社会化办好中俄文化艺术节、朝鲜民族文化艺术节、绥芬河国际口岸贸易博览会、东宁宝玉石文化节等活动。深化与东莞市在人才、产业、园区、对外开放等10个领域合作交流。

　　（四）坚持城乡一体、协同并进，加快构建区域协调发展新局面。协调发展是全面建成小康社会的必然选择。我们要深入落实区域协调发展战略，积极融入哈长城市群和省会城市建设，推动中心城市、内陆县市与口岸深度融合，促进一核隆起、多核联动、一体发展。一是坚决打好城市升级战役。按照精塑江南、提升老城、加快建设智慧城市的部署，着力完善城市功能、提升城市品质、优化城市环境，加快建设有内涵、有风格、有品质的国际现代山水森林城市。优化城市空间格局，高标准启动《城市总体规划（2020－2035年）》修编，做好沿江城市设计和城区控详规划，推进全国新一轮土地利用总体规划试点城市建设。推动老城区功能品位双提升，翻建平安街、新安街等12条主次干道，逐步提高步行和自行车、公交出行分担率，增强城市主干道快速通行能力和支路循环能力；开工建设牡丹江综合交通枢纽，新火车站投入使用；持续提高供电、供热、供水质量，新增天然气用户2万户、停车泊位7000个，提升城市运行保障能力。继续举全市之力建设江南现代新城，加快建设教育、医疗、体育、文化等公共服务设施，高标准推进绿化美化亮化净化工程，引进城市运营商、产业运营商、公共服务运营商，加快推进产城融合发展。推动城市信息化、标准化、精细化管理，构建“智慧+”城市服务体系，建设智慧化城管平台系统；深入开展市容市貌、“僵尸车”等综合整治，全面启动市综合行政执法局工作；继续争创全国文明城市。二是扎实推进新型城镇化和美丽乡村建设。以国家级试点为牵动，因地制宜推进特色小镇建设发展，提升城镇产业支撑能力和吸纳就业能力，改造城关镇、重点镇棚户区5116户；扎实开展农村人居环境整治三年行动，启动新一轮美丽乡村建设，建设美丽乡村示范村20个。三是不断完善区域协调发展机制。建立市县联动发展机制，合理确定功能分区，在园区建设、产业发展上优势互补、资源共享、分工协作、错位发展；各县（市）区都要尽快适应我国推动高质量发展带来的指标、政策、标准、统计体系变化和绩效评价、考评办法的变化，重新考量加快发展的新格局、新方略，确保在全省新一轮评比中继续取得好位次。完善利益分享机制，鼓励各县（市）区为绥东国家重点开发开放试验区、绥芬河综合保税区、牡丹江经开区引进项目。完善合作共建机制，支持森工、农垦改革发展。

　　（五）推进生态文明建设，加快建设美丽牡丹江。生态优势是我市的核心优势。我们要牢固树立绿色发展理念，以打造“两座金山银山”为抓手，加快释放生态建设新优势。一是坚决打好污染防治攻坚战。以“打赢蓝天保卫战”为重点，强化环保责任，加大环境监管执法力度，严格控制工业、城镇生活、农业农村污染物排放，深化水泥、石化等行业集中整治，做好已改造燃煤锅炉后续监管，采取燃料化、肥料化等措施提高秸秆和废弃菌袋综合利用率，推进建成区内黑臭水体治理，完成银龙溪、北安河清淤，餐厨废弃物处置项目投入使用，市区PM2.5年均浓度稳步下降。推进“厕所革命”，新建改扩建旅游厕所45座，改造中心城区旱厕27座，按照政府支持、农民主体、多方参与、试点先行的原则，启动农村改厕工作，力争三年内全市农村“厕所革命”取得明显成效。二是加大生态系统保护和修复力度。全面落实“河长制”、“湖长制”，继续实施饮用水源地综合整治，加快荒沟、龙虎山2个电站建设，力争林海水库获批开工、奋斗水库完成主体工程，推进牡丹江沿江、海浪河2个国家湿地公园建设，全市植树造林8万亩以上，治理水土流失30万亩以上，市区新增绿地20万平方米以上。完成生态保护红线、永久基本农田、城镇开发边界三条控制线划定和自然保护区内矿业权退出、禁养区畜牧生产企业退出、环境恢复治理等工作，确保水生态文明城市建设试点通过国家验收。开展绿色生活“十进”活动，大力倡导简约适度、绿色低碳的生活方式，让牡丹江的天更蓝、山更绿、水更清、空气更清新。

　　（六）切实保障和改善民生，不断提高群众获得感幸福感安全感。我们要始终把人民利益摆在至高无上的地位，既尽力而为，又量力而行，找准民生突出问题及其症结，周密谋划、用心操作，让群众得到看得见、摸得着的实惠。一是坚决打好精准脱贫攻坚战。按照“两不愁、三保障”要求，深入开展产业增收、转移就业、教育扶智、金融扶持、社保政策兜底脱贫攻坚行动，打好“教育+科技+培训+实战+包保”组合拳，全力补齐产业扶贫和饮水安全短板，重点解决好因病因残致贫返贫问题，实施更加严格的督查考核和退出机制，确保脱真贫、真脱贫，切实提高群众对扶贫工作的认可度。二是千方百计推进更高质量和更充分就业。坚持就业优先战略和积极就业政策，特别是注重解决结构性就业问题，坚持政府促进与市场调节相结合，扩大求职创业补贴范围，开发应用“就业地图”，新增城镇就业5.3万人，城镇登记失业率控制在4.3%以内。三是着力加强社会保障。实施全民参保计划，五项社保新增参保3万人，机关事业单位养老保险制度改革全覆盖；提高城乡低保、特困供养标准，构建困难群众“10+1”社会救助体系；提高养老机构服务质量，新增医养结合床位500张；推进保障性住房建设，改造市区棚户区500户，完成全部建档立卡贫困户危房改造，安置回迁居民8000户；紧跟国家多主体供应、多渠道保障、租购并举住房制度政策调整，探索我市实施办法，切实提高住房保障能力。四是大力发展社会事业。优先发展教育事业，深入落实科教兴国战略，深化教育领域综合改革，推动城乡义务教育一体化发展，着力解决中小学生课外负担重、“择校热”、“大班额”等突出问题，解决好婴幼儿照护和儿童早期教育服务问题，提高高中阶段教育普及率，推动职业教育产教融合、校企合作，促进驻牡高校内涵式发展，支持和规范社会力量兴办教育，江南4所新建学校投入使用。推进“健康牡丹江”行动，深化医药卫生体制改革，完善市级公立医院改革配套财政补偿机制和村镇卫生服务体系，建设智慧卫生网络信息平台，抓好心脑血管等重大疾病防治和心理健康知识普及工作，继续实施“中西名医引进计划”，重点在提高引进层次和诊疗水平上下功夫；广泛开展全民健身活动，新建全民健身路径50处，办好“健康雪城”赏冰乐雪系列活动。实施文化惠民工程，加快国家文化消费试点城市建设，建成公共文化数字服务平台，公共文化服务体系基层覆盖率达到90%；做好文物保护利用和文化遗产保护传承，挖掘本土特色文化内涵，培育本土作家群体，鼓励文艺精品创作，叫响地域特色文化品牌。五是打造共建共治共享社会治理格局。完善城乡社会治理体系建设，建立健全“三社联动”工作机制，加快“一体化”综治中心和社会组织市级孵化中心、县（市）区孵化基地建设，推动社会治理重心向基层下移。落实军民融合发展战略，支持驻牡部队建设，做好优抚安置工作。依法管理民族宗教事务，争创“全国民族团结进步示范市”。推进政务诚信、商务诚信、社会诚信和司法公信建设，确保城市信用排名全国领先。健全公共安全体系，提高综合防灾减灾能力，从严从细落实安全责任，深入开展安全生产、食品药品及医疗器械专项整治，切实保障人民群众生命财产安全。继续开展信访积案化解、重大集体访隐患攻坚专项行动，着力解决一批历史遗留问题。深入推进数据警务等“六大工程”，加快立体化、信息化社会治安防控体系建设，始终保持对各类违法犯罪严打高压态势，加快建设“平安牡丹江”。

　　（七）加强政府自身建设，不断提高政府治理能力和水平。打铁必须自身硬。我们要把学习贯彻党的十九大精神作为首要政治任务，扎实推进“两学一做”学习教育常态化制度化，全面开展“不忘初心、牢记使命”主题教育，坚定不移用习近平新时代中国特色社会主义思想统一思想和行动，切实提高“四个意识”、增强“八个本领”，围绕建设现代化牡丹江的宏伟目标，以改革创新激发发展动力，以勤政廉洁凝聚民心群力。一是继续深化重点领域改革。统筹推进政府机构改革，科学配置政府部门及内设机构权力、明确职责；持续深化“放管服”改革，强化权力、责任、中介服务、公共服务、负面“五个清单”动态管理，完善市行政审批局功能，实现“一枚印章管审批”；加快建设“互联网+后台”的“4.0政府”，完善网上审批服务中心和网上民生服务中心功能，让企业和群众办事更加便捷；加快镜泊湖管理体制改革，深化市经开区水、气、热、地、运输、信息服务等供应方式市场化改革，推进温春镇省经济发达镇行政管理体制改革试点。二是持续推进法治政府建设。严格执行政府重大决策法定程序，重大事项和重大决策部署落实情况及时向市委请示报告；深入落实法治政府建设“实施方案”，做好政府立法工作，全面落实行政执法责任制，加大行政执法监督力度，打造“双随机一公开”信息监管平台，开展联合检查试点工作；拓宽群众参政渠道，加快政务公开和信息化建设，自觉接受人大、政协和社会监督，高质量办好提案议案；扎实推进“七五”普法，加快建设“法治牡丹江”。三是全面加强作风和廉政建设。持之以恒整治“四风”，深入查摆带有“新表现”的形式主义和官僚主义，严格执行中央八项规定实施细则和省委改进工作作风密切联系群众的规定，大力倡导“五个好作风”、争做“三个好把式”，领导干部带头到基层和一线宣讲政策、服务群众，努力凝聚全面振兴发展的强大合力。严格落实党风廉政建设“一岗双责”，严守“六大纪律”；象百姓过日子一样精打细算，严控“三公”经费支出，努力打造节约型政府。深入落实“宁静工程”和“门禁制度”，严厉查处破坏发展环境案件，坚决打好整顿作风优化发展环境攻坚战。

　　各位代表，万里征程风正劲,千钧重任再扬帆。让我们更加紧密地团结在以习近平同志为核心的党中央周围，在市委的正确领导下，凝聚起全市人民的磅礴力量，不忘初心、牢记使命，锐意进取、埋头苦干，朝着建设富强民主文明和谐美丽现代化牡丹江的宏伟目标奋勇前进！
